# Real-World Effectiveness of Bempedoic Acid in Reducing LDL-C: An Electronic Medical Records-Based Analysis

**DOI:** 10.1016/j.jacadv.2026.102726

**Published:** 2026-04-06

**Authors:** Julius L. Katzmann, Christian Becker, Tobias F. Schneider, Konstantin Articus, Gereon Keuken, Ulrich Laufs

**Affiliations:** a Klinik und Poliklinik für Kardiologie, Universitätsklinikum Leipzig, Leipzig, Germany; b Daiichi Sankyo Europe GmbH, Munich, Germany; c IQVIA Commercial GmbH & Co. OHG, Frankfurt am Main, Germany

**Keywords:** cardiovascular prevention, drug utilization, lipid-lowering therapy, real-world data

**What is the clinical question being addressed?**

Is the real-world effectiveness of bempedoic acid in reducing low-density lipoprotein cholesterol comparable to that observed in clinical trials?

**What is the main finding?**

Based on electronic medical records, bempedoic acid reduced low-density lipoprotein cholesterol depending on concomitant lipid-lowering therapy to a similar extent as in clinical trials.

In addition to statins and ezetimibe, bempedoic acid (BA) has emerged as a third orally available drug with proven cardiovascular benefit for lowering low-density lipoprotein cholesterol (LDL-C). In randomized controlled trials, BA reduced LDL-C compared to placebo by 17.4% to 18.1% in patients on maximally tolerated statin and by 21.4% to 28.5% in patients with statin intolerance.^1^

The achieved absolute reduction in LDL-C determines the cardiovascular benefit of an LDL-C–lowering drug. The real-world effectiveness of lipid-lowering therapies (LLTs) depends on the extent to which clinical trial findings can be replicated in everyday care. Deviations could be due to differences in comorbidities, concomitant medication, medication adherence among treated patients, and the general set-up of a clinical trial which may include more frequent visits in contrast to usual care.

In this study, we analyzed the LDL-C reduction following BA prescription using electronic medical records available in the IQVIA Disease Analyzer database that contains representative demographic, diagnostic, and prescription data collected in general and specialized practices in Germany. Patients were included if BA was initiated between November 2020 and June 2023, and if they had at least 1 recorded LDL-C measure 6 months before BA initiation and between 15 days and 6 months afterward. As only retrospective anonymized data were analyzed, no ethics committee approval was required.

A total of 1,032 patients from 848 general practices and 49 cardiologist practices in Germany were identified. Patients had a mean age of 66.3 (SD: 10.4) years, 42.4% were females. Atherosclerotic cardiovascular disease was present in 77.8% of patients with 65.1% coronary artery disease, 23.0% cerebrovascular disease, and 23.9% peripheral artery disease. Diabetes was present in 23.3% and hypertension in 72.7%. According to the 2021 European Society of Cardiology prevention guidelines, 82.9% of patients had very-high cardiovascular risk. Baseline LDL-C was 127.5 (51.5) mg/dL.

In 425 of the 1,032 patients, BA was initiated in addition to no or unchanged concomitant LLT. The median (IQR) relative LDL-C reductions were as follows (Figure 1).

• No concomitant LLT (n = 54): 29.9% (26.3%)
• Unchanged concomitant LLT (n = 371): 20.3% (30.7%)
  ◦ Concomitant LLT with statin (n = 255): 16.0% (29.5%)
  ◦ Concomitant LLT without statin (n = 116): 31.5% (28.2%)

**Figure 1:**
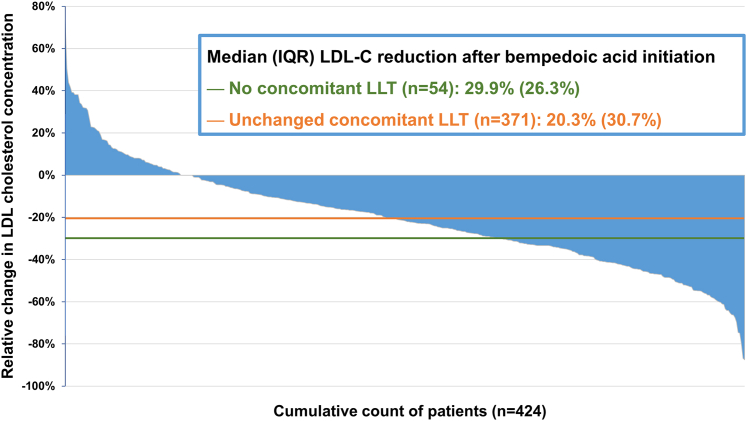
**Change in Low-Density Lipoprotein Cholesterol Following Bempedoic Acid Initiation** Waterfall plot for the change in LDL-C concentration following bempedoic acid initiation in patients without or unchanged concomitant LLT. One patient whose LDL-C change exceeded ±5 SDs from the mean is not shown. LDL-C = low-density lipoprotein cholesterol; LLT = lipid-lowering therapy.

This translated into absolute reductions in LDL-C of median (IQR) 49.6 (48.0) mg/dL, 19.0 (39.1) mg/dL, 13.0 (28.2) mg/dL, and 40.2 (45.5) mg/dL, respectively, in the 4 groups.

In the remaining patients, concomitant LLT changed from the first and second measurement time point. Among them, 156 patients initiated ezetimibe and BA simultaneously, encompassing “true” new initiations and possibly, treatment restarts or switches. The median (IQR) relative LDL-C reductions in these patients were as follows.

• All ezetimibe starters (n = 156): 34.0% (41.0%)
  ◦ Pretreatment with statin (n = 101): 31.7% (38.3%)
  ◦ No pretreatment with statin (n = 55): 35.8% (31.0%)

This translated into absolute reductions in LDL-C of median (IQR) 37.5 (67.0) mg/dL, 31.0 (50.0) mg/dL, and 57.0 (81.0) mg/dL, respectively, in the 3 groups.

In 130 patients, BA and ezetimibe were simultaneously initiated, and statin therapy was terminated. These patients had median (IQR) relative and absolute LDL-C reductions of 30.3% (41.5%) and 34.0 (71.0) mg/dL.

Published real-world evidence on the effectiveness of BA outside of clinical trials is scarce. In a previous monocenter retrospective study of 73 patients, BA reduced LDL-C by 36.7% after <3 months, declining to 20.3% after 12 months.^2^ To the best of our knowledge, the current report adds data from the largest real-world cohort on the effectiveness of BA to date.

The observed relative reductions in LDL-C following BA prescription were similar to or even exceeded those reported in clinical trials. The average relative and importantly, absolute reduction in LDL-C was highest in patients who were not taking LLT, and lower when BA was added to pre-existing LLT, particularly a statin. The median relative LDL-C reductions were in part larger than the mean reductions. This reflects the heterogeneity of treatment effect (Figure 1) and is likely attributable to differences in medication adherence that may be lower in real-world practice than in clinical trials and interindividual treatment response.

In international expert panel position papers^3^ and the 2025 focused update of the 2019 European Society of Cardiology/European Atherosclerosis Society guidelines for the management of dyslipidemias,^4^ treatment with BA is recommended for patients unable to take statins and for those not achieving the LDL-C target with the maximally tolerated statin dose and ezetimibe. These recommendations reflect common clinical scenarios in which BA may be considered to achieve the LDL-C goal before the treatment with proprotein convertase subtilisin/kexin type 9 inhibitors, for which access is still restricted. The approach of an oral triple combination LLT with a statin, ezetimibe, and BA is expected to substantially increase LDL-C goal achievement to 61.8% of high- and very-high-risk patients, as indicated by a recent simulation study that sequentially employed a statin intensification step, addition of ezetimibe, and addition of BA, and was based on the multinational SANTORINI cohort.^5^ The similarity between the expected relative LDL-C reduction based on clinical trial results that was used in that and other simulation studies, and the observed real-world LDL-C reductions suggests that the simulation studies likely provide meaningful information.

In conclusion, this study based on electronic medical records confirms the effectiveness of BA in reducing LDL-C concentration in a real-world setting.

## Funding support and author disclosures

Dr Katzmann has received honoraria and/or travel grants from 10.13039/100001003Boehringer Ingelheim, 10.13039/501100022274Daiichi Sankyo, 10.13039/100004312Lilly, 10.13039/100004336Novartis, and Synlab, outside of this work. Dr Becker is an employee of 10.13039/501100022274Daiichi Sankyo. Dr Articus is an employee of 10.13039/501100022274Daiichi Sankyo. Keuken is an employee of IQVIA. Dr Laufs has received speaker honoraria or consulting fees from 10.13039/100002429Amgen, Apontis, 10.13039/100004325AstraZeneca, 10.13039/100004326Bayer, Berlin-Chemie, 10.13039/100001003Boehringer Ingelheim, 10.13039/501100022274Daiichi Sankyo, 10.13039/100004312Lilly, MSD, 10.13039/100004336Novartis, NovoNordisk, 10.13039/100004319Pfizer, Sanofi, and Synlab, outside of this work. All other authors have reported that they have no relationships relevant to the contents of this paper to disclose.

## References

[1] Ballantyne C.M., Bays H., Catapano A.L., Goldberg A., Ray K.K., Saseen J.J. (2021). Role of bempedoic acid in clinical practice. Cardiovasc Drugs Ther.

[2] Warden B.A., Cardiology B.-A., Purnell J.Q., Duell P.B., Fazio S. (2022). Real-world utilization of bempedoic acid in an academic preventive cardiology practice. J Clin Lipidol.

[3] Banach M., Penson P.E., Farnier M. (2023). Bempedoic acid in the management of lipid disorders and cardiovascular risk. 2023 position paper of the International lipid expert panel (ILEP). Prog Cardiovasc Dis.

[4] Mach F., Koskinas K.C., van Roeters Lennep J.E. (2025). 2025 focused update of the 2019 ESC/EAS guidelines for the management of dyslipidaemias. Eur Heart J.

[5] Katzmann J.L., Ray K.K., Lee B. (Published online December 23, 2025). Oral triple combination lipid-lowering therapy and LDL cholesterol goal attainment: a simulation using the SANTORINI study cohort. Eur J Prev Cardiol.

